# Altered functional connectivity associated with striatal dopamine depletion in Parkinson’s disease

**DOI:** 10.1093/texcom/tgad004

**Published:** 2023-02-20

**Authors:** Atsushi Shima, Rika Inano, Hayato Tabu, Tomohisa Okada, Yuji Nakamoto, Ryosuke Takahashi, Nobukatsu Sawamoto

**Affiliations:** Department of Neurology, Kyoto University Graduate School of Medicine, Kyoto 606-8507, Japan; Department of Neurosurgery, Kyoto University Graduate School of Medicine, Kyoto 606-8507, Japan; Department of Neurology, Kyoto University Graduate School of Medicine, Kyoto 606-8507, Japan; Department of Diagnostic Imaging and Nuclear Medicine, Kyoto University Graduate School of Medicine, Kyoto 606-8507, Japan; Department of Diagnostic Imaging and Nuclear Medicine, Kyoto University Graduate School of Medicine, Kyoto 606-8507, Japan; Department of Neurology, Kyoto University Graduate School of Medicine, Kyoto 606-8507, Japan; Department of Neurology, Kyoto University Graduate School of Medicine, Kyoto 606-8507, Japan; Department of Human Health Sciences, Kyoto University Graduate School of Medicine, Kyoto 606-8507, Japan

**Keywords:** cortico-basal ganglia circuit, fMRI, PET, striatum, subthalamic nucleus

## Abstract

We aimed to clarify whether dopamine depletion in the posterior dorsal striatum in early-stage Parkinson’s disease (PD) alters synchronized activity in the cortico-basal ganglia motor circuit. In sum, 14 PD patients and 16 matched healthy controls (HC) underwent [11C]-2-β-carbomethoxy-3-β-(4-fluorophenyl) tropane positron emission tomography to identify striatal dopamine-depleted areas. The identified map was applied to functional magnetic resonance imaging (fMRI) to discover abnormalities in functional connectivity (FC) during motor-task and rest-state in PD patients in the drug-off state relative to HC. Striatal dopamine-depleted areas formed synchronized fMRI activity that largely corresponded to the cortico-basal ganglia motor circuit. Group comparisons revealed that striatal dopamine-depleted areas exhibited decreased FC with the medial premotor cortex during motor-task and with the medial, lateral premotor and primary motor cortices during rest-state. Striatal dopamine-depleted areas also elucidated decreased FC in the subthalamic nucleus (STN) in PD both during motor-task and rest-state. The STN regions that exhibited reduced FC with striatal dopamine-depleted areas demonstrated excessive FC with the lateral premotor and primary motor cortices in PD only during rest-state. Our findings suggest that striatal dopamine-depleted area reduced synchronized activity with the motor cortices and STN, which, in turn, induces an abnormal increase in coupling between the areas in PD.

## Introduction

Parkinson’s disease (PD) is neuropathologically characterized by progressive loss of heterogeneous populations of neurons including neuromelanin laden dopamine neurons in the pars compacta of the substantia nigra (Brissaud 1925). Within the pars compacta, the degree of dopamine neuronal loss tends to be most prominent in the ventrolateral tier, resulting in regional depletion of striatal dopamine particularly in the posterior dorsal subdivisions of the striatum. In vivo imaging studies have reported that the level of dopamine depletion in the putamen correlates with the severity of motor symptoms (Bernheimer et al. 1973; Seibyl et al. 1995; Vingerhoets et al. 1997; Benamer et al. 2000; Wang et al. 2007). However, the relationship between dopamine depletion and motor symptoms in PD has not been fully clarified.

One of the earliest proposed models for this relationship is the “firing rate model”, which is based on the observation of tonic activity changes in basal ganglia neurons in l-methyl-4-phenyl-1,2,3,6-tetrahydropyridine (MPTP)-treated primate Parkinsonism. This model predicts that dopamine depletion in the striatum results in increased activity in the indirect pathway via over-inhibition of the external globus pallidum (GPe), disinhibition of the subthalamic nucleus (STN), and decreased activity in the direct pathway. The net effect is an increase in inhibitory activity of basal ganglia output neurons in the internal globus pallidum (GPi) and substantia nigra pars reticulata (SNr) over thalamocortical neurons (Bergman et al. 1994). Although this model played an important role in the development of deep brain stimulation (DBS) therapy, it has received recent criticism. For instance, later studies demonstrated only small increases in GPi firing in the Parkinsonian state (Levy et al. 2002; Hutchison et al. 2004). Further, the model is difficult to reconcile with clinical findings that lesions in the motor thalamus relieved, rather than worsened, motor symptoms in PD (Pan et al. 1996; Duval et al. 2006).

Another proposed model is the “oscillation model,” which is based on the analysis of temporal activity patterns in basal ganglia neurons in PD (Brown et al. 2001; Brown 2006). This model proposes that dopamine depletion leads to excessively synchronized oscillatory activity in motor areas of the STN and GPi, which contributes to motor symptoms in PD. This concept was motivated by the electrophysiological observation that exaggerated oscillatory activity, particularly in the beta band in the STN during the rest or idling state, is suppressed by dopamine replacement therapy, and the degree of suppression correlates with the levels of improvement in bradykinesia and rigidity (Weinberger et al. 2006; Brown 2007). Excessively synchronized activity appears to occur both locally in the basal ganglia nuclei and globally in the subcortical and cortical areas. These abnormalities in cortico-basal ganglia circuits require clarification as the basal ganglia do not send direct output to motor pathways but cause motor symptoms indirectly via the circuit (Brown 2006). In addition, the link between excessively synchronized activity and striatal dopamine depletion requires elucidation given the recent evidence that dopamine modulates basal ganglia function at extra-striatal sites, and abnormal modulation at these sites may contribute to PD symptoms (Rommelfanger and Wichmann 2010; Furukawa et al. 2022). Moreover, because exaggerated oscillatory activity observed in the STN is suppressed during voluntary movement in PD, the abnormalities in the circuits during movement as well as rest need further clarification.

The aim of the present study was to clarify whether dopamine depletion in the posterior dorsal subdivisions of the striatum alters temporally synchronized activity in the cortico-basal ganglia circuit during movement and rest in PD. Dopamine-depleted areas in the striatum were identified using [11C]-2-β-carbomethoxy-3-β-(4-fluorophenyl) tropane-positron emission tomography ([11C]-CFT-PET) in patients with PD. The identified map was applied to functional magnetic resonance imaging (fMRI) to detect abnormalities in functional connectivity (FC) of the dopamine-depleted areas or altered spatial patterns of correlated activity across the network during motor-task and rest-state in patients with PD in the drug-off state compared with that in healthy controls (HC). Patients with early stages of the disease were recruited to minimize effects related to motor fluctuations and non-dopaminergic symptoms.

## Materials and methods

### Participants

In total, 14 patients diagnosed with PD according to the clinical diagnostic criteria and 16 age- and sex-matched HC participated (Table 1). All of the participants were assessed as right-handed according to the Edinburgh Handedness Inventory (Oldfield 1971; Postuma et al. 2015). None of the participants reported a history of any neurological or psychiatric disorders other than PD. The participants were also screened for cognitive impairments using the Mini-Mental State Examination and Frontal Assessment Battery. Ten patients received medication for PD (levodopa equivalent dose, 275.6 ± 179.9 mg/day) (Tomlinson et al. 2010). Four patients did not receive any medication. All of the participants provided written informed consent in accordance with the dictates of the Ethics Committee (approval number #C-408), Graduate School and Faculty of Medicine, Kyoto University and the Declaration of Helsinki and its subsequent revisions.

**Table 1:** Disease severity of Parkinson’s disease patients.

Group	*n* (male)	Age	Mini-mental state examination	Frontal assessment battery	Time from symptom onset (years)	H&Y	UPDRS total score (sum of part I, II, III, and IV)	UPDRS part III
PD	14 (7)	64.0 ± 10.0	29.5 ± 0.9	16.1 ± 1.6	3.6 ± 2.6	1.8 ± 0.9	28.4 ± 11.8	17.2 ± 7.1
HC	16 (7)	62.3 ± 5.1	29.5 ± 1.3	15.6 ± 1.6	-	-	-	-

Data are presented as mean ± SD. PD, Parkinson’s disease; HC, healthy controls; H&Y, Hoehn and Yahr stage; UPDRS, Unified Parkinson’s Disease Rating Scale.

H&Y and UPDRS were assessed during practically defined off-state.

### Behavioral measures

All PD patients were assessed in a practically defined off-state after withdrawal of levodopa for at least 12 h or after withdrawal of dopamine agonists and amantadine for at least 24 h. The symptoms of PD were evaluated according to the Unified Parkinson’s Disease Rating Scale (UPDRS) part III (Fahn et al. 1987). Six patients each were classified as tremor dominant type and postural instability and gait disturbance type, respectively (Jankovic et al. 1990).

### [11C]-CFT-PET data acquisition

All participants underwent [11C]-CFT-PET to assess striatal dopamine transporter availability (Frost et al. 1993). Image acquisition was performed using GE Advance Tomograph (GE/Yokogawa, Tokyo, Japan) with the inter-slice septa retracted. Head movements during the scan were minimized with an elastic headband restraint. In total, 35 slice images with 4.25-mm inter-slice spacing were acquired. PET images were obtained in four sequential frames over 20 min (4 × 300 s time frames), starting 60 min after a bolus intravenous injection of 10-mCi [11C]-CFT. The images were individually corrected for effects of radiation attenuation using a rotating 68-Ge source after [11C]-CFT data acquisition. All PET scanning was performed within 1 month after MRI examinations (Ishii et al. 2016).

### MRI data acquisition

MRI images were acquired using Siemens MAGNETOM Trio 3 Tesla MR System (Germany) equipped with an eight-channel phased-array head coil in a practically defined off-state after withdrawal of levodopa for at least 12 h or dopamine agonists and amantadine for at least 24 h.

Blood oxygen level-dependent (BOLD) fMRI was performed in an axial orientation with the following parameters: repetition time (TR) = 3000 ms; echo time (TE) = 30 ms; flip angle (FA) = 90°; field of view (FOV) = 192 × 192 mm^2^; slices = 48, voxel size = 3 × 3 × 3 mm^3^ (Yamao et al. 2023). The two initial scans were discarded, and the subsequent 203 scans were obtained for each session. Data acquisition in each session occurred over a period of 10 min and 9 s and involved 21-s blocks of 15 rest conditions alternating with blocks of 14 finger-to-thumb opposition movements. During the rest period, participants fixed on a white cross presented on the screen and remained motionless. During the tasks period, participants fixed on a white circle on the screen and performed self-initiated movements at a rate around 1 Hz: (i) index finger to thumb opposition movement of the right hand or (ii) left hand. One session each was conducted for the right- and left-hand movement, respectively. Prior to scanning, participants practiced the movements until they were able to perform them at a suitable speed (Oguri et al. 2013).

High-resolution structural images (1 × 1 × 1 mm^3^) were acquired using magnetization-prepared rapid gradient echo (TR = 2000 ms; TE = 4.38 ms; FA = 8°; FOV = 176 × 192 mm; slices  = 160). Field-map images in an axial orientation were collected at 3 × 3 × 3 mm resolution using a gradient echo sequence (TR = 511 ms; TE1/TE2 = 5.19/7.65 ms; flip angle = 60°; FOV =  192 × 192 mm; slices = 46).

### Flipping of [11C]-CFT-PET and MRI images

Based on the laterality of decreases in [11C]-CFT binding and motor symptoms, all PET and MRI images were orientated such that the left side of the striatum corresponded to lower [11C]-CFT binding in patients with PD. Data of four patients with right dominant decrease of striatal [11C]-CFT binding were left-right flipped for further analysis. In HC, images of five randomly selected subjects were flipped to control for the effects of handedness on fMRI activity. The number of movements during fMRI scans was matched between the groups in each side determined after flipping (hand movement contralateral to right-side striatum: PD 17.1 ± 4.9, HC 17.6 ± 4.8, *P* = 0.75; hand movement contralateral to left-side striatum: PD 17.5 ± 5.2, HC 17.4 ± 4.7, *P* = 0.51).

### Definition of regions of interest in striatal dopamine-depleted areas

The index of specific [11C]-CFT binding to dopamine transporter was estimated as a (region-cerebellum)/cerebellum ratio at 60–80 min post-injection (Laakso et al. 1998). For the reference region, circular regions of interest (ROIs) of 31.2 mm in diameter were defined in each cerebellar hemisphere in five contiguous planes with Analyze software (version 11.0, Biomedical Imaging Resource, Mayo Foundation, Rochester, MN, USA) (Sawamoto et al. 2008). Specific [11C]-CFT binding images were transformed to the individual T1 space using the rigid body registration matrix from the [11C]-CFT images integrated into T1-weighted images. Transformed specific-binding images were spatially normalized to Montreal Neurological Institute (MNI) 2-mm space based on anatomical information from the T1-weighted images and nonlinear registration tool (FNIRT) in FMRIB Software Library (FSL) version 5.0.7 (Smith et al. 2004; Anderson et al. 2007).

The transformed PET images were analyzed with FMRI Expert Analysis Tool version 6.00 in FSL. The images were smoothed with a Gaussian kernel with a full-width half-maximum of 5 mm and statistically analyzed using a general linear model. Patients with PD and HC were compared using two-group unpaired *t*-tests. Statistical maps were thresholded at *P* < 0.05 (familywise-error corrected over the whole brain).

The regions of interest in striatal dopamine-depleted areas (StrROIs) were defined as voxels with significantly decreased binding in [11C]-CFT PET in the PD group and were contained within gray matter regions in individual T1-weighted images. Clusters of decreased binding in the MNI space were inversely transformed to individual T1 space. The StrROIs were subsequently delineated as striatal gray matter regions using T1-weighted images with FMRIB’s Integrated Registration and Segmentation Tool (FIRST) and transformed into individual fMRI space using affine registration implemented with FMRIB’s Linear Image Registration Tool (FLIRT; Jenkinson et al. 2002; Patenaude et al. 2011).

### FC analysis of StrROIs on each phase

BOLD fMRI images were processed using tools from FSL (https://fsl.fmrib.ox.ac.uk/fsl/fslwiki/). Head movements were corrected with MCFLIRT and non-brain areas were removed using Brain Extraction Tool (Jenkinson et al. 2002; Smith 2002). The images were unwarped with gradient-echo based field-map images using FUGUE and spatially smoothed with a Gaussian kernel with a full width half-maximum of 5 mm. Grand-mean intensity of the entire 4D data set was normalized using a single multiplicative factor, and high-pass temporal filtering was applied (Gaussian-weighted least-squares straight line fitting with sigma = 50.0 s) (Jenkinson 2003).

Motor-task FC was calculated as correlations during the concatenated time series of the motor task after removing the rest periods. Rest-state FC was also computed as correlations during the concatenated time series of the rest excluding the task periods. Furthermore, as a comparison, we estimated combined correlations using whole time series involving both motor-task and rest-state.

A voxel-wise analysis was conducted to identify FC across the entire brain between each voxel within the brain and characteristic time series of the StrROI. Representative activity of the StrROI was extracted by calculating average time series in the ROI. Characteristic activity of white matter and cerebrospinal fluid was extracted by measuring the average time series in ROIs, which were specified using FMRIB’s Automated Segmentation Tool and subsequently transformed into individual fMRI space using affine registration (Zhang et al. 2001). Time series of white matter, cerebrospinal fluid, and six head movement parameters were included as covariates of no interest in order to regress out fMRI signals unrelated to cortical neuronal activity. After regressing out these eight nuisance time series, the correlation scores between the time series in each voxel and those of the StrROI were calculated.

Individual correlation maps of motor-task FC, rest-state FC, and combined FC using whole time series were transformed from functional space into individual anatomical space with FIRST and subsequently into MNI 152 2-mm space with FNIRT. The MNI space correlation maps were subjected to a group general linear model with a fixed-effects approach using multi-session and multi-subject analysis. Group comparisons between PD patients and HC were performed using unpaired *t*-tests. Statistical maps were thresholded at *P* < 0.05 (family wise-error corrected over the whole brain).

## Results

### Areas of decreased [11C]-CFT binding within the striatum in Parkinson’s disease

**Fig. 1:**
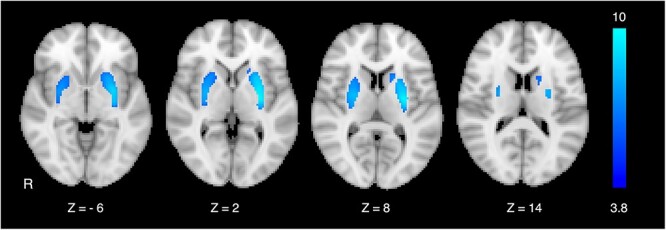
Areas of decreased [11C]-CFT binding within the striatum in PD patients compared with that in HC. Group comparison revealed decreased binding in the posterior dorsal subdivisions of the striatum, whereas binding was relatively preserved in the anterior ventral subdivision, especially in the less affected hemisphere (i.e. right) in PD patients. Statistical parametric maps were superimposed on Montreal neurological institute (MNI) 152 standard-space T1-weighted average structural template images. Values under the MRI slices indicate MNI coordinates. Values on the color bar represent z-scores in this and other related figures. R, the participant’s right side.

[11C]-CFT binding was observed in the anterior ventral subdivision of the striatum wider area on the right side relative to that on the left in patients with PD. In contrast, symmetric binding was observed across the whole striatum in HC. Group comparison revealed that [11C]-CFT binding in the PD group was decreased in the posterior dorsal subdivisions of the striatum to a greater spatial extent in the more affected hemisphere (i.e. left) (*P* < 0.05, corrected; Fig. 1). In contrast, no areas of binding were decreased in HC when compared with PD patients. Areas with significantly decreased [11C]-CFT binding in the PD group were used to generate StrROI for fMRI analyses.

### FC organized by StrROIs in PD and HC groups

**Fig. 2:**
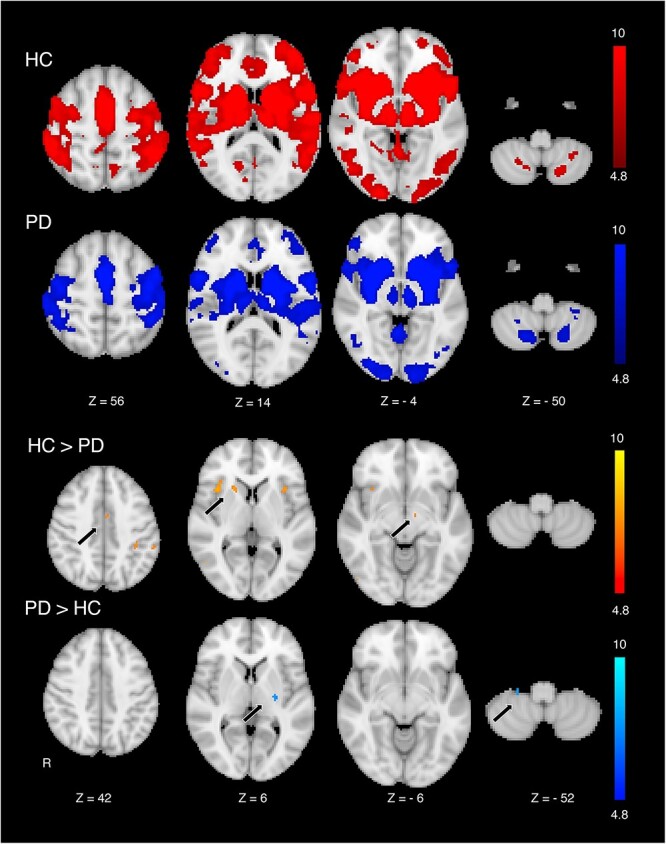
FC mapped in HC and PD patients, organized by striatal areas of decreased [11C]-CFT binding in PD patients using motor-task fMRI. Dopamine-depleted areas in the striatum formed temporally synchronized fMRI activity with cortical areas, predominantly motor cortices including the medial and lateral premotor cortex, primary motor cortex, and primary somatosensory cortex; subcortical areas encompassing the Gpe, Gpi, STN, thalamus, and cerebellum in both HC and PD patients. Group comparison of FC organized by striatal dopamine-depleted areas exhibited decreased FC with the medial premotor cortex and STN and striatal dopamine-preserved areas in the anterior subdivision of the less affected hemisphere in PD patients compared with that in HC. In contrast, striatal dopamine-depleted areas demonstrated excessive synchronization within the posterior striatum in the more affected hemisphere and with the cerebellar cortex in PD patients compared with that in HC.

StrROIs demonstrated significant motor-task FC with broad areas in the bilateral frontal cortex, predominantly in motor-related areas including the medial and lateral premotor cortex, primary motor cortex, and primary somatosensory cortex in both PD patients and HC (*P* < 0.05, corrected; Fig. 2). Significant FC was also observed bilaterally in subcortical areas in the striatum, GPe, GPi, STN, thalamus, and cerebellum in both groups (*P* < 0.05, corrected).

StrROIs exhibited lower motor-task FC in the PD group in the medial premotor cortex compared with the HC group (*P* < 0.05, corrected; Fig. 2). FC in the PD group was also reduced in the anterior striatal area in the less affected hemisphere and in the STN. In contrast, FC of the StrROIs was higher in the posterior striatum in the more affected hemisphere and motor regions in cerebellar cortex than that in the HC group (*P* < 0.05, corrected).

**Fig. 3:**
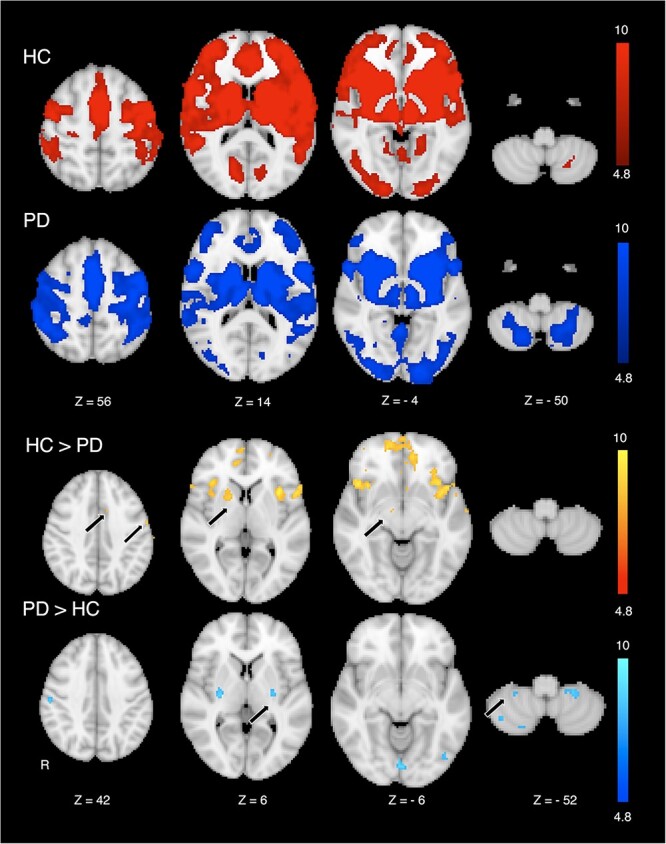
FC mapped in HC and PD patients, organized by striatal areas of decreased [11C]-CFT binding in PD patients using rest-state fMRI. Dopamine-depleted areas in the striatum formed temporally synchronized fMRI activity with motor related areas in both HC and PD patients. Group comparison of FC organized by striatal dopamine-depleted areas exhibited decreased FC with the medial, lateral premotor, and primary motor cortices, STN, and anterior subdivision of the striatum in PD patients compared with that in HC. In contrast, striatal dopamine-depleted areas demonstrated excessive synchronization within the posterior striatum and with the cerebellar cortex in PD patients compared with that in HC.

We next assessed rest-state FC and obtained generally similar results. StrROIs demonstrated significant rest-state FC with broad areas predominantly in motor-related areas in both PD patients and HC (*P* < 0.05, corrected; Fig. 3). Significant FC was also observed bilaterally in subcortical areas in both groups (*P* < 0.05, corrected). StrROIs exhibited lower rest-state FC in the PD group in the medial, lateral premotor, and primary motor cortices compared with the HC group (*P* < 0.05, corrected; Fig. 3). FC in the PD group was also reduced in the anterior striatal area in the less affected hemisphere and in the STN. In contrast, FC of the StrROIs was higher in the posterior striatum and motor regions in cerebellar cortex than that in the HC group (*P* < 0.05, corrected).

Further, we estimated combined FC using whole time series and found generally similar results (*P* < 0.05, corrected; Supplementary Fig. 1).

### FC analysis of ROIs within the STN with reduced connectivity to striatal dopamine-depleted areas

**Fig. 4:**
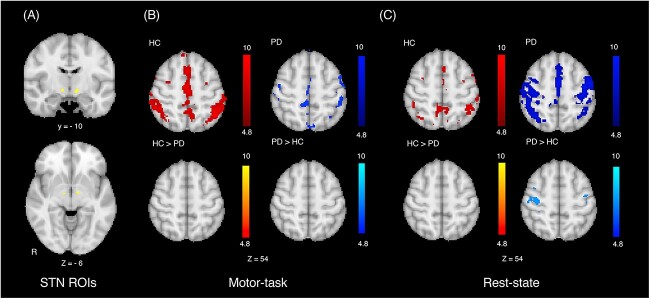
A seed in the STN exhibiting reduced connectivity with striatal dopamine-depleted areas in the PD patients (A) and its FC mapped in HC and PD patients using motor-task fMRI (B) and rest-state fMRI (C). Significant FC of the STN region was observed during motor-task with the medial premotor and parietal cortices in HC and with the medial, lateral premotor, and primary motor cortices in PD patients. Similar FC was found during rest-state, though distribution was smaller in HC and was larger in PD patients. Group comparison revealed that FC of STN regions was not significantly different during motor-task, whereas FC during rest-state was significantly higher with the lateral premotor and primary motor cortices in PD patients than in HC.

Post hoc seed-based FC analysis was performed using the ROI in the STN, which demonstrated significantly reduced FC of the StrROI in the PD group compared with that in the HC group calculated during the analysis using whole time series (*P* < 0.05, corrected; Fig. 4A). This post hoc analysis was conducted given that the STN is a target for stereotactic surgery and is a key structure for alleviation of motor symptoms in PD.

By placing a seed in the STN based on FC analysis of the StrROI, correlation maps of FC were obtained for the PD and HC groups. Significant FC of the STN region was observed during motor-task with the medial premotor and parietal cortices in the HC group and with the medial, lateral premotor, and primary motor cortices in the PD group (*P* < 0.05, corrected; Fig. 4B). Group comparison revealed no significant difference in the motor cortices. Motor-task FC of the STN region was significantly higher in the lower midsagittal cerebellum in the PD group compared with that in the HC group although significant motor-task FC of the STN region was not observed in the area in individual group analyses in the PD nor the HC (*P* < 0.05, corrected; Supplementary Fig. 2).

We next explored rest-state FC. Similar FC was found during rest-state, though distribution was smaller in the HC group and was larger in the PD group compared with motor-task FC. Group comparison revealed that rest-state FC of the STN region was significantly higher in the lateral premotor and primary motor cortices in the PD group compared with that in the HC group (*P* < 0.05, corrected; Fig. 4C). Rest-state FC of the STN region was also higher in the lower midsagittal cerebellum in the PD group compared with that in the HC group, although significant rest-state FC of the STN region was not found in the area in individual group analyses in the PD nor the HC (P < 0.05, corrected; Supplementary Fig. 2). We then computed interaction between group and fMRI condition (motor-task and rest-state) and found that the interaction did not reach a statistically significant level. Finally, we estimated combined FC using whole time series and found similar results to rest-state FC (P < 0.05, corrected; Supplementary Fig. 3).

## Discussion

The present study demonstrated that [11C]-CFT binding in patients with early stage PD was decreased in the posterior dorsal subdivisions of the striatum, whereas the binding was relatively preserved in the anterior ventral subdivision, especially in the less affected hemisphere (i.e. right) (Brooks et al. 1990; Lee et al. 2000). Dopamine-depleted areas in the striatum exhibited temporally synchronized fMRI activity with cortical areas, predominantly the motor cortices including the medial and lateral premotor cortex, primary motor cortex, and primary somatosensory cortex; and subcortical areas encompassing the GPe, GPi, STN, thalamus, and cerebellum both during motor-task and rest-state in PD patients and HC. The synchronized FC network largely corresponded to motor subdivisions of the cortico-basal ganglia circuit, supporting the view that aberrant neuronal processing in this circuit underlies motor symptoms in PD. Group comparison revealed that striatal dopamine-depleted areas exhibited decreased FC with the medial premotor cortex during motor-task and with the medial, lateral premotor and primary motor cortices during rest-state. Striatal dopamine-depleted areas also elucidated decreased FC with striatal dopamine-preserved areas in the anterior subdivision of the less affected hemisphere in PD patients and in the STN both during motor-task and rest-state. In contrast, striatal dopamine-depleted areas demonstrated excessive synchronization within the posterior striatum to a greater extent in the more affected hemisphere and with the cerebellar cortex. Moreover, the STN regions that exhibited reduced FC with striatal dopamine-depleted areas demonstrated excessively synchronized activity with the lateral premotor and primary motor cortices in PD patients only during rest-state. Collectively, the present findings suggest that striatal dopamine-depleted area reduced synchronized activity with the motor cortices and STN and increased locally synchronized activity within the posterior striatum both during motor-task and rest-state. This, in turn, induces an abnormal increase in coupling between the STN and the lateral premotor and primary motor cortices during rest-state in patients with PD when compared with HC.

Dopamine-depleted regions in the posterior dorsal subdivisions of the striatum exhibited significantly reduced FC with the medial, lateral premotor, and primary motor cortices during rest-state in the drug-off state in patients with PD when compared with HC, consistent with a previous study using resting-state fMRI (Ruppert et al. 2020). We further demonstrated that striatal dopamine-depleted areas elucidated decreased FC with the medial premotor cortex during motor-task. The posterior putamen, anatomically defined as the striatal region posterior to the anterior commissure, also exhibited decreased FC with sensorimotor cortices in the drug-off state in early stages of PD using resting-state fMRI (Helmich et al. 2010; Luo et al. 2014). In contrast, increased FC between the anterior striatum and somatosensory cortical regions in patients with PD has been reported using resting-state fMRI. This discrepancy may be attributed to regional differences in dopamine depletion within the striatum (Yu et al. 2013). Indeed, dopamine-depleted areas in the posterior striatum exhibited decreased FC with the anterior striatum in the less affected hemisphere in PD patients both during motor-task and rest-state where dopamine was observed to be relatively preserved in this study and a previous report (Bell et al. 2015). These findings agree with a previously proposed hypothesis that cortico-striatal FC behave differently in dopamine-depleted posterior striatum and relatively dopamine-preserved anterior striatum in the less affected hemisphere in PD patients (Helmich et al. 2010).

Decreased FC between striatal dopamine-depleted areas and STN both during motor-task and rest-state was observed in patients with PD. The regions located laterally within the STN correspond to motor subdivisions (Prodoehl et al. 2008). The present findings support the hypothesis that striatal dopamine depletion modulates the sensitivity of the striatum to cortical inflow, thereby altering striatal control of activity in the indirect pathway (Magill et al. 2001; Tseng et al. 2001; Sharott et al. 2017; West et al. 2018). This change has been proposed to induce excessive beta burst in the STN particularly during rest.

The STN regions identified as territories with decreased FC with dopamine-depleted striatal areas exhibited abnormal temporal synchronicity of fMRI activity with the lateral premotor and primary motor cortices in patients with PD during rest-state. Anatomically defined STN regions also demonstrated higher fMRI FC with the lateral premotor and primary motor cortices in the drug-off state in patients with PD compared with that in HC using resting-state fMRI (Baudrexel et al. 2011; Kurani et al. 2015). In addition, the present findings align well with electrophysiological studies which have reported increased power and coherence of beta frequency oscillatory activity in the STN and frontal cortex in the drug-off state and rest in animal models and patients with PD (Sharott et al. 2005; Fogelson et al. 2006; Litvak et al. 2011; Hirschmann et al. 2013). Moreover, the present findings are compatible with fMRI results demonstrating normalized FC between the STN and motor cortices in the dopaminergic drug-on state in PD patients compared with that in HC (Mathys et al. 2016). The present findings showing abnormal coupling between the STN and motor cortices during rest-state but not during motor-task imply that the coupling underlies pathophysiology of akinesia or poverty of spontaneous movement and rigidity rather than bradykinesia or slowness of a performed movement. Other PD motor manifestations such as smaller movements than desired should be investigated in future studies.

Dopamine-depleted areas within the posterior striatum demonstrated excessive local FC in patients with PD both during motor-task and rest-state in this study. In dopamine-depleted regions in the rat striatum, increased firing rates and abnormally synchronized firing at beta frequencies were observed in a widespread population of spiny projection neurons constituting the indirect pathway but not in spiny projection neurons of the direct pathway during cortical activation corresponding to an awake, behaving state. (Sharott et al. 2017). Computational models have predicted that beta oscillations in PD originate within networks of striatal neurons (McCarthy et al. 2011; Damodaran et al. 2015; Kondabolu et al. 2016). Intraoperative recordings of patients with PD undergoing DBS surgery revealed that spiny projection neurons in the striatum fired at higher frequencies involving the beta band with abundant spike burst when compared with patients with essential tremor and isolated dystonia (Singh et al. 2016). These lines of evidence suggest that excessive local fMRI FC in the posterior striatum observed in the present PD patients reflects overactive, abnormally synchronized activity of spiny projection neurons predominantly constituting the indirect pathway, which influences the generation of beta oscillations in basal ganglia circuits.

The present study identified abnormal decoupling between dopamine-depleted striatal areas and STN regions, as well as excessive synchronization between STN regions and motor cortices during rest-state in PD patients. These findings are in accordance with the notion that striatal dopamine depletion modifies activity in the indirect pathway (Magill et al. 2001; Sharott et al. 2017; West et al. 2018), leading to perturbed synchronized activity between the STN and motor cortices. Excessive oscillatory activity in the STN was suppressed by blocking glutamatergic afferents, which are thought to arise mainly from the cortex via the so-called hyperdirect pathway (Tachibana et al. 2011; Wilson 2015). Optogenetic approaches were used to selectively stimulate afferent STN fibers from the motor cortex at a high frequency of 130 Hz (similar to DBS), which improved motor disturbances in an animal model of PD (Gradinaru et al. 2009). These findings support the view that increased STN and motor cortex synchronicity is mediated at least partly via the hyperdirect pathway. Collectively, these findings suggest that under striatal dopamine depletion, control of activity in the indirect and hyperdirect pathways collapse, resulting in abnormal synchronized oscillatory activity in the motor cortices and STN as proposed by the “dynamic activity model” (Nambu et al. 2015).

Dopamine-depleted striatal areas exhibited increased FC with motor regions in the cerebellum both during motor-task and rest-state in PD patients in this study. FC between the posterior putamen and cerebellum in patients with PD was reported to be increased in the drug-off state and was normalized to levels comparable to those in HC following levodopa administration using resting-state fMRI (Simioni et al. 2016). One possible interpretation of the increased FC between the posterior putamen and cerebellum is that it arises from compensatory mechanisms (Wu and Hallett 2013). In contrast, the STN regions that exhibited reduced FC with striatal dopamine-depleted areas demonstrated excessively synchronized activity with the lower midsagittal cerebellum both during motor-task and rest-state in the PD group compared with that in the HC group, although significant FC of the STN region was not observed in the cerebellar area in individual group analyses in the PD nor the HC. Thus, interpretation of the present results was not straightforward, and this point should be further investigated.

In this study, network FC across different brain regions was evaluated by calculating temporal coupling of slow fluctuations (< 0.1 Hz) of fMRI BOLD signals. We, thus, assessed cardinal motor features of PD involving akinesia and rigidity rather than transient symptoms such as a difficulty to initiate movement. BOLD signals are preferentially sensitive to local field potentials, which correlate most strongly with gamma band neuronal synchronization (Logothetis et al. 2001; Fox et al. 2007; Engel et al. 2013). In addition, BOLD variance can be explained by independent contributions of beta and alpha band power (Scheeringa et al. 2011). Therefore, the FC observed in the present study was likely to be influenced by gamma band neural synchronization as well as beta and alpha frequency ranges. This assumption should be tested in studies using simultaneous electroencephalogram fMRI acquisition.

The present study has limitations. First, because of the limited availability of the PET scanning, the number of subjects was relatively small. Second, rest-state FC in this study was estimated using concatenated time series during rest periods between motor-task block rather than using continuous resting-state fMRI. Rest-state FC, therefore, was not attributable purely to rest and can involve the preparation for the motor-task (Fair et al. 2007). Third, significantly higher FC between the STN and motor cortices was observed in PD patients than HC not during motor-task but during rest-state, though interaction of group and fMRI condition (motor-task and rest-state) did not reach a statistically significant level. Therefore, further studies are needed to clarify whether the excessive FC in PD patients occurs specifically during rest or not. Fourth, we did not evaluate FC under dopaminergic treatment since the treatment effects may be complex including not only normalization on dopamine-depleted area but also over-stimulation on dopamine-preserved area.

Dopamine-depleted areas in the striatum exhibited decreased FC with motor cortices and STN and increased FC within the posterior striatum in PD patients both during motor-task and rest-state in this study. In addition, the STN regions that demonstrated reduced FC with striatal dopamine-depleted areas exhibited abnormal temporal synchronicity of fMRI activity with the lateral premotor and primary motor cortices only during rest-state in PD patients. These findings highlight novel therapeutic targets for DBS, including the putamen (Montgomery et al. 2011). Future studies should clarify the key factors linking striatal dopamine depletion and impaired control of activity in the indirect and hyperdirect pathways in PD patients.

## Supplementary Material

Supplementary_Figure_221103sawa_tgad004

Sup_1_tgad004

Supp2_tgad004

Supp3_tgad004

## Data Availability

The data that support the findings of this study are available from the corresponding author upon reasonable request.

## CRediT authors statement

Atsushi Shima (Conceptualization, Data curation, Formal analysis, Investigation, Project administration, Writing—original draft, Writing—review and editing), Rika Inano (Data curation, Formal analysis, Funding acquisition, Investigation, Methodology), Hayato Tabu (Conceptualization, Data curation, Formal analysis, Investigation, Methodology, Project administration, Resources, Writing—review and editing), Tomohisa Okada (Conceptualization, Data curation, Investigation, Project administration, Writing—review and editing), Yuji Nakamoto (Writing—review and editing), Ryosuke Takahashi (Project administration, Writing—review and editing), Nobukatsu Sawamoto (Conceptualization, Data curation, Formal analysis, Funding acquisition, Investigation, Methodology, Project administration, Supervision, Writing—original draft, Writing—review and editing).
