# Supplementary material for: Altered functional connectivity associated with striatal dopamine depletion in Parkinson’s disease
Source: Cereb Cortex Commun. 2023 Feb 20;4(1):tgad004. doi: 10.1093/texcom/tgad004 (PMC10026073; doi:10.1093/texcom/tgad004)
Supplement: Supplementary_Figure_221103sawa_tgad004 [file supplementary_figure_221103sawa_tgad004.docx]

**Supplementary Figures**

**Supplementary Figure1.**

Functional connectivity mapped in healthy controls (HC) and Parkinson’s disease (PD) patients, organized by striatal areas of decreased [11C]-CFT binding in PD patients using whole time series fMRI. Dopamine-depleted areas in the striatum formed temporally synchronized fMRI activity with motor related areas in both HC and PD patients. Group comparison exhibited decreased functional connectivity with motor cortices, STN and anterior subdivision of the striatum in PD patients compared to that in HC. In contrast, striatal dopamine-depleted areas demonstrated excessive synchronization within the posterior striatum and with the cerebellar cortex in PD patients compared to that in HC.

**Supplementary Figure2.**

Functional connectivity of the seed in the STN identified as reduced connectivity region with striatal dopamine-depleted areas in the Parkinson’s disease (PD) group, mapped in the cerebellum in healthy controls (HC) and PD patients using motor-task fMRI (A) and rest-state fMRI (B).

**Supplementary Figure3.**

Functional connectivity of the seed in the STN exhibiting reduced connectivity with striatal dopamine-depleted areas in the Parkinson’s disease (PD) group using whole time series fMRI. The STN regions that exhibited reduced functional connectivity with striatal dopamine-depleted areas in PD patients demonstrated significant functional connectivity with motor cortices in these patients. Group comparison revealed that functional connectivity of STN regions with motor cortices was significantly higher in the PD group than in the healthy control group.
