# Supplementary figures and images for: Altered functional connectivity associated with striatal dopamine depletion in Parkinson’s disease
Source: Cereb Cortex Commun. 2023 Feb 20;4(1):tgad004. doi: 10.1093/texcom/tgad004 (PMC10026073; doi:10.1093/texcom/tgad004)

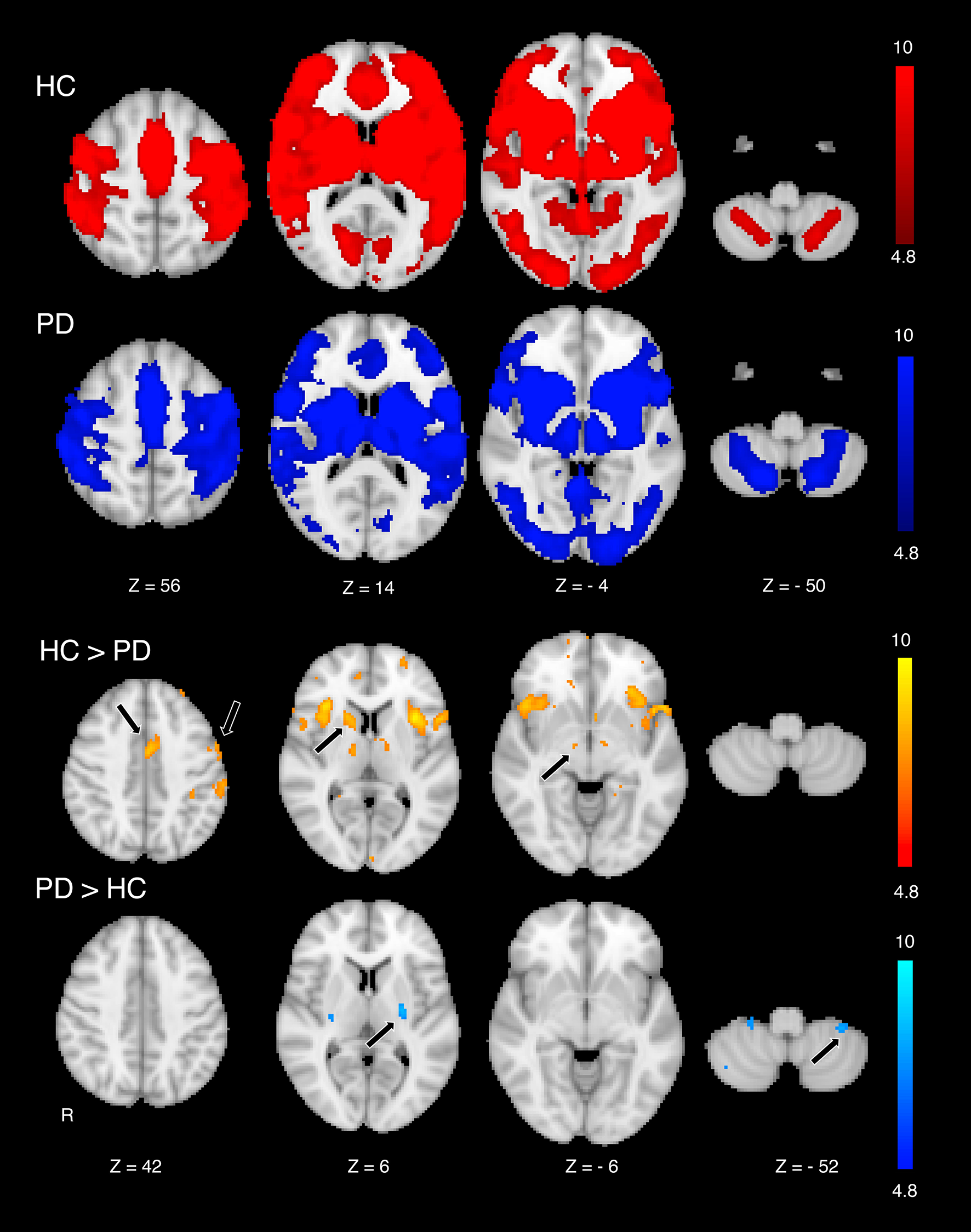

Supplement: Sup_1_tgad004 [file sup_1_tgad004.zip › Sup_1_tgad004.tif]

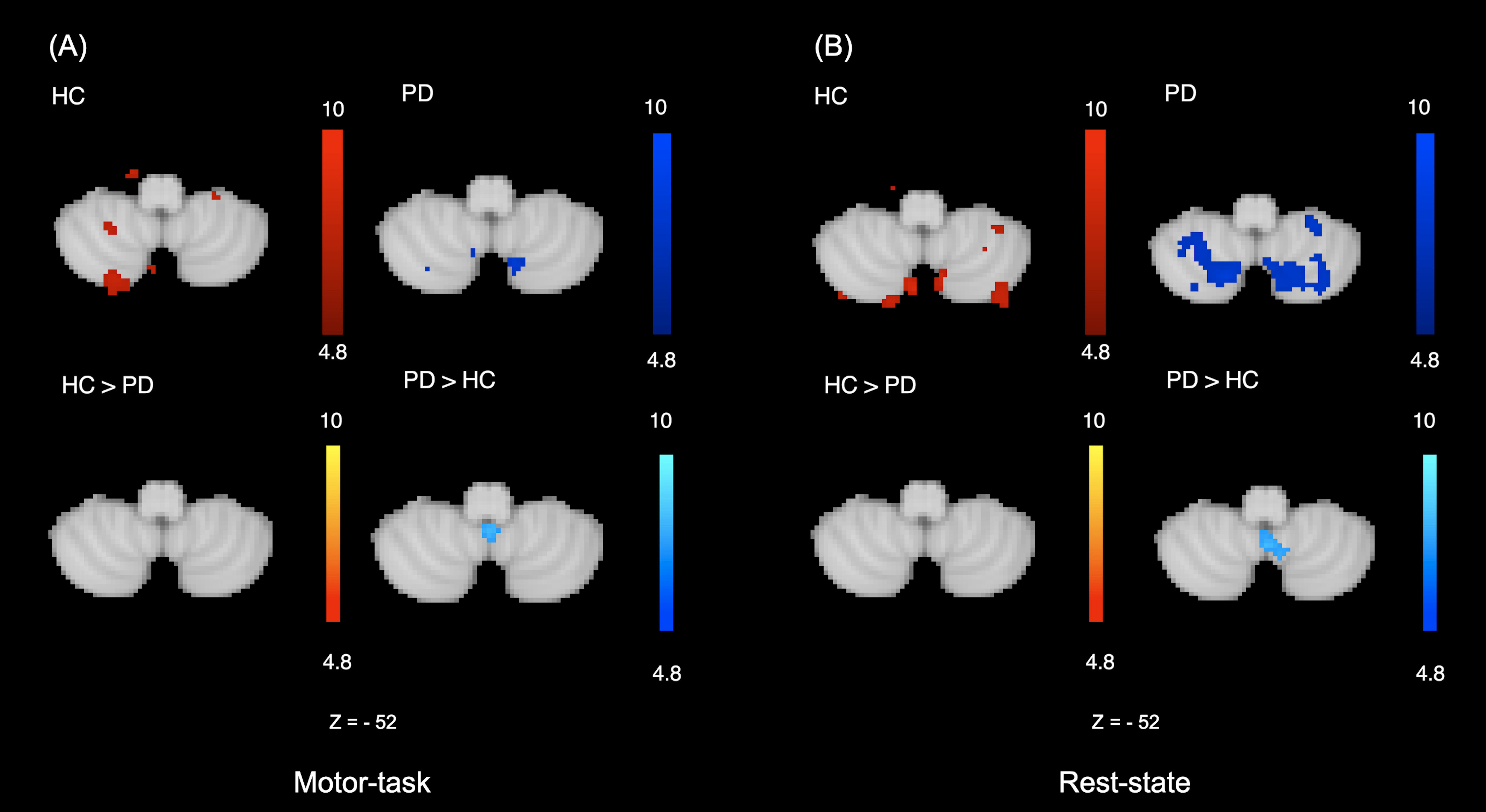

Supplement: Supp2_tgad004 [file supp2_tgad004.zip › Supp2_tgad004.tiff]

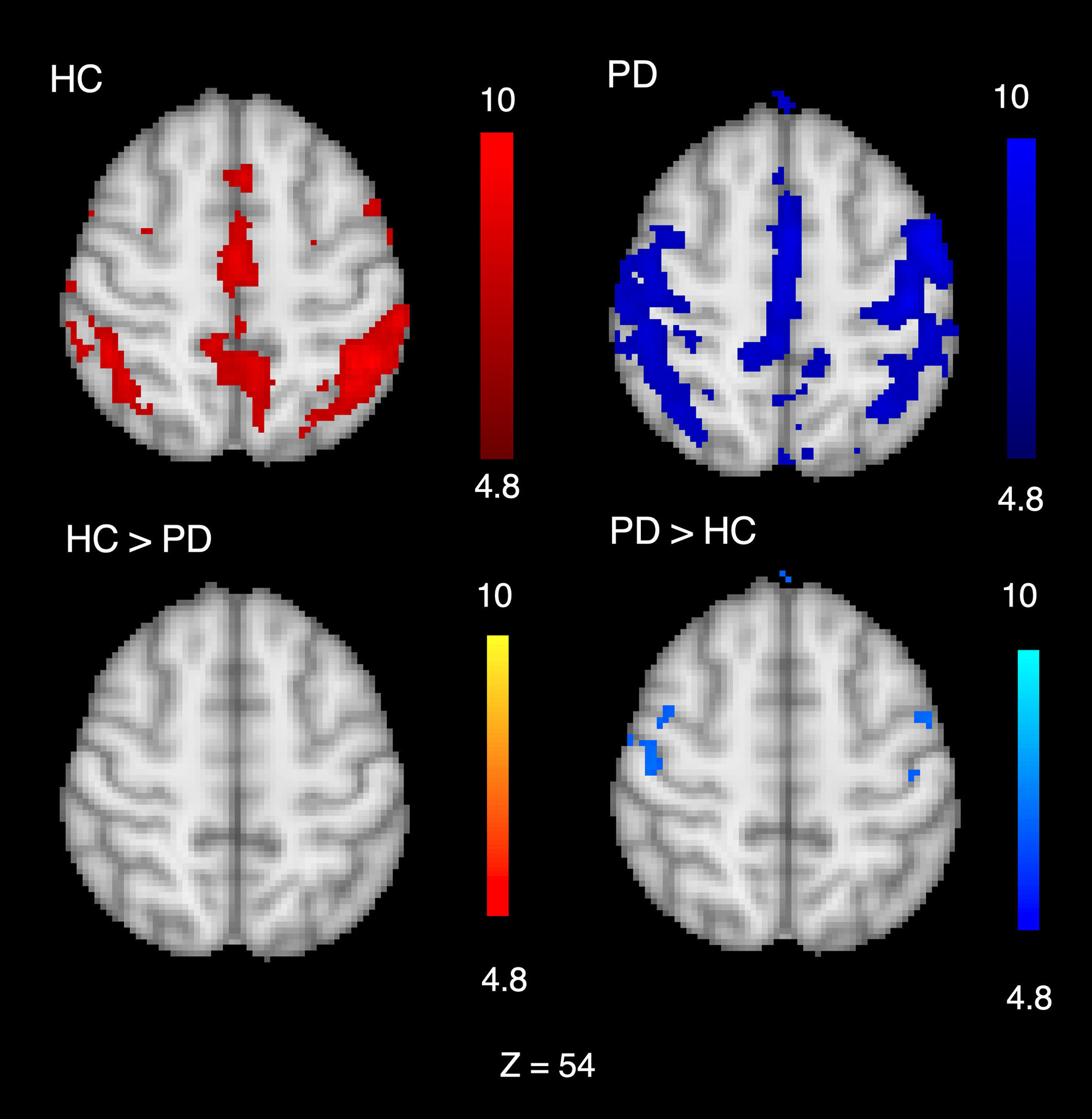

Supplement: Supp3_tgad004 [file supp3_tgad004.zip › Supp3_tgad004.tif]
